# Ethnic Differences in Glucose Homeostasis Markers between the Kyushu-Okinawa Population Study and the Framingham Offspring Study

**DOI:** 10.1038/srep36725

**Published:** 2016-11-10

**Authors:** Hiroaki Ikezaki, Masumi Ai, Ernst J. Schaefer, Seiko Otokozawa, Bela F. Asztalos, Katsuyuki Nakajima, Yanhua Zhou, Ching-Ti Liu, Paul F. Jacques, L. Adrienne Cupples, Norihiro Furusyo

**Affiliations:** 1 Cardiovascular Nutrition Laboratory, Human Nutrition Research Center on Aging at Tufts University and Tufts University School of Medicine, 711 Washington Street, Boston, MA 02111 USA; 2 Nutritional Epidemiology Program, Human Nutrition Research Center on Aging at Tufts University and Tufts University School of Medicine, 711 Washington Street, Boston, MA 02111 USA; 3 Department of General Internal Medicine, Kyushu University Hospital, 3-1-1 Maidashi, Higashi-ku, Fukuoka 812-8582 Japan; 4 Department of Insured Medical Care Management, Tokyo Medical and Dental University Hospital, 1-5-45 Yushima, Bunkyo-ku, Tokyo 113-8510 Japan; 5 Department of Public Health, Sapporo Medical University School of Medicine, S1 W17, Chuo-ku, Sapporo 060-8556 Japan; 6 Department of Clinical Laboratory Medicine, Gunma University, 3-39-22 Showa-machi, Maebashi, Gunma 371-8511 Japan; 7 Department of Biostatistics, Boston University School of Public Health, 801 Massachusetts Avenue CT3, Boston, MA 02118 USA

## Abstract

We compared markers of glucose homeostasis and their association with diabetes and impaired fasting glucose (IFG) in Fukuoka, Japanese subjects (n = 1108) and age-, gender- and menopausal status-matched participants in the Framingham Offspring Study (n = 1096). The markers examined included fasting glucose, insulin, adiponectin, and glycated albumin, as well as body mass index (BMI), use of medications, and history of diabetes. The results showed that IFG prevalence in Japanese men (15.9%) and women (7.4%) were 50% less than those observed in Framingham men (34.5%) and women (21.4%) (*P* < 0.001). However, the diabetes prevalence in Japanese men at 13.3% was twice as high (*P* < 0.01) as the rate in Framingham men at 6.5%, while these rates were similar in women. Median insulin levels in Japanese men (4.6 μIU/mL) and women (4.3 μIU/mL) were about 50% lower (*P* < 0.001) than those in Framingham men (10.8 μIU/mL) and women (9.9 μIU/mL), as were insulin resistance values (*P* < 0.001). These population differences were also observed after subjects were stratified by glucose levels. In conclusion, our data indicate that there is significantly less IFG, lower insulin levels, and insulin resistance, but higher diabetes prevalence in Fukuoka men than in Framingham men, indicating that insulin deficiency may be an important cause of diabetes in Japan.

It has been reported that the prevalence of diabetes mellitus appears to be higher in Asian populations than in Caucasians^1^,^2^,^3^,^4^,^5^. Since modern hominids have only lived in Europe and Asia for approximately the past 60,000 years, the ethnic and population differences between the Japanese and Caucasians are most probably related to differences in the environment over many generations, although different genotypes may have been selected for. It has been suggested that Japanese people may have somewhat more visceral adipose tissue at any given level of body mass index (BMI) associated with enhanced liver fat deposition and pro-inflammatory cytokine production than Caucasians^6^,^7^,^8^,^9^. Studies have also indicated that when homeostasis model assessment of insulin resistance (HOMA-IR) values are assessed, Asian-American people have significantly lower values than those observed in Caucasians^10^,^11^,^12^,^13^. Recently Ahuja and colleagues compared 298 American white men and 294 Japanese men in Japan aged 40–49 years, and documented significantly lower HOMA-IR values in Japanese men than their Caucasian counterparts, even after adjustment for BMI and fasting glucose^14^. They concluded that the higher glucose disposition index in Caucasian men, even after adjustment for visceral adipose tissue, may explain the lower diabetes susceptibility of Caucasians as compared to Japanese people^14^. We hypothesized that there may be ethnic differences in the relationship between glucose intolerance and insulin sensitivity. Our goal was to compare the markers of glucose homeostasis associated with diabetes and impaired fasting glucose (IFG) in men and women participating in a population study in Fukuoka, Japan with age and gender matched participants from the Framingham Offspring Study.

## Results

The results of our studies in female participants in both populations are shown in Table 1. The age and menopausal status between the two populations were virtually identical by selection. Females in Fukuoka had significantly lower values for BMI, waist circumference, prevalence of being overweight or obese, and prevalence of IFG. However the prevalence rates of diabetes were similar at 4.2% for Fukuoka and 4.4% for Framingham (*P* = 0.98). Women in Fukuoka had significantly lower values for fasting glucose, insulin, HOMA-IR, homeostasis model assessment of β cell function (HOMA-β), and adiponectin than did Framingham women. However, levels of glycated albumin (GA) were significantly higher in Fukuoka women than in their Framingham counterparts. After adjustment for BMI these significant population differences in fasting insulin, HOMA-IR, HOMA-β, adiponectin and GA were still present (data not shown). Therefore despite substantially lower levels of BMI and measures of insulin resistance, the Japanese women did not have less diabetes or higher adiponectin levels as would have been expected from their BMI values. In fact the Fukuoka women had significantly lower adiponectin levels than their Framingham counterparts even after BMI adjustment.

The results of our studies in male participants in both populations are shown in Table 2. The mean age between the two populations was identical by design. Similar to the women, men in Fukuoka had significantly lower values for BMI, waist circumference, prevalence of being overweight or obese, and prevalence of IFG. Interestingly the prevalence rates of diabetes were significantly higher for Fukuoka men at 13.3% versus 6.5% for Framingham men (*P* < 0.01). Moreover men in Fukuoka also had significantly lower values for fasting insulin, HOMA-IR, and HOMA-βthan Framingham men. Moreover, levels of GA were significantly higher in Fukuoka men than in their Framingham counterparts. After adjustment for BMI these significant population differences in fasting insulin, HOMA-IR, HOMA-β and GA were still present, and the values of adiponectin were significantly lower in Fukuoka men than in their Framingham counterparts (data not shown). Therefore like the Japanese women, Fukuoka men, despite having substantially lower BMI values and measures of insulin resistance, did not have less diabetes or higher adiponectin levels, as one would have been expected based on their substantially lower BMI values. They, in fact, had twice the prevalence rates of diabetes than men of the same age in Framingham.

Female subjects in both populations were stratified by glucose homeostasis status into normal glucose tolerance (NGT), IFG, or diabetes groups based on their history of diabetes or fasting glucose levels using standard American Diabetes Association (ADA) criteria as shown in Table 3. The same differences observed in the entire population, were also observed in these three groups with regard to BMI, being overweight or obese, waist circumference, fasting plasma glucose, insulin, and HOMA-IR. Japanese women had significantly lower values for all of these parameters as compared to Framingham women. In contrast, GA levels were significantly higher in NGT and IFG Fukuoka women than in their Framingham counterparts. These differences were not seen in the diabetes groups, where values were similar. Interestingly the insulin levels in Fukuoka women with diabetes at 6.2 μIU/mL were less than 40% of the values observed in Framingham women with diabetes at 16.3 μIU/mL. Moreover the insulin levels in Fukuoka women with diabetes were significantly lower than those values in Framingham NGT women (*P* < 0.001) and values for HOMA-IR in Fukuoka women with diabetes were similar to Framingham NGT women.

Male subjects in both populations were stratified by glucose homeostasis status into NGT, IFG, or diabetes groups as shown in Table 4. The same differences observed in the entire population, were also observed in these three groups with regard to BMI, being overweight or obese, waist circumference, fasting plasma insulin, HOMA-IR, and HOMA-β. Japanese men had significantly lower values for all of these parameters as compared to Framingham men. Adiponectin levels were similar between populations in the NGT group, significantly higher in Framingham men in the IFG group, but significantly lower in Framingham men in the diabetes group as compared to Fukuoka men. In addition values for GA were significantly higher in Fukuoka men than in Framingham men, but only in the IFG group. Interestingly the insulin levels in Fukuoka men with diabetes at 6.6 μIU/mL were less than 40% of the values observed in Framingham men with diabetes at 19.0 μIU/mL. Moreover the insulin levels in Fukuoka men with diabetes were significantly lower than those values in Framingham NGT men (*P* < 0.001), similar to the population differences in women. In addition median values for HOMA-IR in Fukuoka men with diabetes were identical to Framingham NGT men.

Table 5 shows the results of age- and BMI-adjusted multivariable logistic regression analysis model for presence of diabetes classified by sex and population. In this model, we examined the effects of GA per 1% change, insulin per 1 μIU/mL change, and adiponectin per 1 μg/mL change. In this analysis GA was important in all groups with regard to a significant association with diabetes, adiponectin was important in Fukuoka women and Framingham men, and insulin was important in Fukuoka men.

However since GA is strongly related to diabetes we reran these analyses after removing GA. Table 6 shows the results of the second age- and BMI-adjusted multivariable logistic regression analysis model for presence of diabetes classified by sex and population. In this analysis adiponectin was significant in all groups except Fukuoka men, while insulin was only significant in Fukuoka men.

## Discussion

This population based study directly compared the prevalence rates of diabetes, IFG, and markers of glucose homeostasis in both men and women in the free-living state in Japan and the United States. A strength of this study is its relatively large sample size, and the fact that all biochemical assays were carried out in the same laboratory by the same technical staff using the same assays. While many endocrinologists and epidemiologists invoke insulin resistance as being critical for the pathogenesis of diabetes, the data presented here indicate that insulin deficiency also plays a critical role in causing diabetes mellitus, especially in the Japanese population. Surprisingly Japanese women and men with diabetes not only had much lower insulin levels than Framingham counterparts with diabetes, but also had significantly lower levels than their NGT counterparts in Framingham. In addition insulin resistance (HOMA-IR) values were the same in Japanese women and men with diabetes as in their NGT counterparts in Framingham.

Previous studies including the United States National Health Interview Survey (1997–2008) and the Center for Disease Control, indicated Asian population including Japanese living in the United States had higher prevalence of diabetes than did Caucasians^1^,^2^,^3^,^4^,^5^. One potential reason of ethnic differences in the prevalence of diabetes is adoption of the western lifestyle. Kawate and colleagues examined the prevalence of diabetes in Japanese migrants and their offspring on the island of Hawaii as compared to Japanese subjects living in Hiroshima^15^. They found that diabetes was significantly more common in subjects in Hawaii than in Japan. Their studies indicated similar caloric intake between the groups, but consumption of animal fat and simple carbohydrates (sucrose and fructose) were at least twice as high in subjects in Hawaii as in Japan. Moreover lower estimated physical activity was observed in the subjects in Hawaii as compared to the subjects in Japan. These data undergo the importance of lifestyle including dietary habits for development of diabetes. Our data indicate that native Japanese men in the free living state have a higher prevalence rate of diabetes than do their United States counterparts. We did not see such differences in women, but we did observe the same population differences with regard to insulin levels. It is important to emphasize that the median ages of our populations were 55 years in men and 53 years in women. Based on data from the Japanese Ministry of Health, Labour, and Welfare, the prevalence of diabetes in Japanese men and women in their 50 s were 10.6% and 6.5%, while for men and women in their 60 s it was 20.1% and 13.3%, and for men and women in their 70 s or older it was 22.3% and 17.0%^16^. Therefore while insulin levels were low in Japanese men and women compared to their Caucasian counterparts, the reason for the gender differences in diabetes prevalence may relate to the fact that our populations were in their fifties, and we might have also seen differences in women if their median ages had been greater.

The major purpose of this study was to directly compare and investigate the ethnic difference in pathogenesis of diabetes in both populations in Japan and the United States. Of note is that HOMA-IR values for both female populations increased when one compares the NGT and IFG groups, but did not increase further in the diabetes group in Fukuoka, in contrast to Framingham. Moreover HOMA-β values progressively decreased in Framingham women when one compares values in the NGT, IFG, and diabetes groups, while in Fukuoka women the major decline was only in the diabetic group. These data indicate that especially in Japanese women β cell dysfunction plays a key role in the pathogenesis of diabetes. In addition HOMA-IR values in both male populations increased when one compares the NGT, IFG, and diabetes groups. Moreover HOMA-β values progressively decreased in both male populations when one compares to values in the NGT, IFG, and diabetes groups. However in all groups these values were about 50% lower in the Japanese population than in Framingham. These data indicate that both insulin resistance and β cell dysfunction (lower HOMA-β) play a key role in the pathogenesis of diabetes.

Jensen and colleagues evaluated β cell function in four US ethnic groups (African-Americans, Asian-Americans, Caucasians, and Hispanic Americans). They reported that both insulin resistance and impaired β cell function were important in determining glucose disposal in all ethnic groups^10^. Torrens and colleagues assessed insulin sensitivity and β cell function in non-diabetes premenopausal or early peri-menopausal non-Hispanic white women, as well as in African American, Chinese American, Japanese American, and non-Mexican-American Latino women. Values for insulin resistance (HOMA-IR) were significantly lower in African Americans, Chinese Americans, and Japanese Americans when compared with non-Hispanic white women even after adjustment for waist circumference and impaired fasting glucose. In addition Japanese American women had the lowest levels of plasma insulin as compared to other groups, similar to what we have observed in our study^11^. A strength of our study was that the insulin assay used in was the same for both populations. Moreover the assay was performed in the same laboratory using the same automated platform by the laboratory personnel. Kodama and colleagues identified 74 studies that measured the insulin sensitivity index and acute insulin response to glucose in three major ethnic groups: Africans, Caucasians, and East Asians^13^. The investigators noted that the African cohorts had the greatest insulin resistance and the highest insulin response to glucose, while the converse was true for the East Asian cohorts, with the Caucasian groups being in the middle. These data show that both insulin resistance and deficiency are important in the pathogenesis of adult onset diabetes. Moreover these data indicate that insulin deficiency plays a major role in developing diabetes in Japanese population. One proposed concept to explain these ethnic differences in insulin response is genetic variation among races. Recently, genome-wide association studies found that genetic variation at the *KCNQ1, UBE2E2*, or *C2CD4A/B* genes were associated with diabetes in Japanese subjects^17^,^18^. Although many of these genes seem related to insulin deficiency, whether or not variants in these genes could explain impaired β cell function remains unclear. Further studies to clarify the relationship between genetic variants at these gene loci and β cell function are necessary.

Adiponectin is also known to play an important role in modulating insulin sensitivity with lower levels being associated with obesity, insulin resistance, diabetes, and excess CVD risk^19^. Kadowaki and colleagues compared 98 American white men and 92 Japanese men in Japan aged 40–49 years, and documented that Japanese men had significantly (*P* < 0.001) lower adiponectin levels than their Caucasian counterparts, even though the Japanese men had a much lower prevalence of obesity^20^. These data are consistent with our own findings. Our data indicate that adiponectin levels were not elevated in the Japanese population despite their significantly lower BMI values as compared to Framingham. These population differences in adiponectin levels, despite a lower prevalence of obesity, may in part be due to genetic differences, since about 40% of Japanese subjects have been noted to have an unfavorable adiponectin genotype^21^. Difference in visceral adipose tissue distribution and its ratio to subcutaneous adipose tissue might be another explanation^7^,^8^. It may not just be the amount of body fat, but also the type of body fat that is important in determining adiponectin levels. Khoo and colleagues studied ethnicity, metabolic parameters and the development of diabetes in a multiethnic Asian population^22^. They reported an inverse relationship between adiponectin and BMI and insulin resistance. They also reported women had higher adiponectin levels than did men. We saw similar relationships with BMI in our populations, with elevated BMI being associated with diabetes, increased insulin, and increased insulin resistance, as well as decreased adiponectin levels. In addition adiponectin levels decreased as one looks at the data in those with normal glucose metabolism, as compared to those with IFG and diabetes.

In addition levels of GA were significantly, but modestly greater in Japanese men and women than their Framingham counterparts. When assessed by subgroups of glucose homeostasis, these difference were only found in the Japanese women who had either NGT or IFG, and in the Japanese men who had IFG, as compared to Framingham subjects. These data do suggest that the Japanese subjects have a lower ability to control blood glucose levels especially chronically than their Caucasian counterparts. In our view these differences may relate to a decreased ability to produce sufficient insulin for tight glucose control.

Our study had some limitations. One limitation of our study is its cross-sectional design. Because this was not a prospective study, we were unable to conclude that the insulin deficiency observed in the Japanese population was causal for the excess diabetes observed in Japanese men. In addition, our study lacks oral glucose tolerance data for analysis, thus we were unable to detect patients with either impaired glucose tolerance or diabetes based on an oral glucose load. It has been reported in Asian people that those in the early stages of diabetes or glucose intolerance are only detected with an oral glucose tolerance test^23^,^24^. Therefore we might have detected a number of subjects who were only diabetic based on OGTT criteria. Another shortcoming or limitation is that the samples were collected some 8-9 years apart; however if sample degradation were an issue, one would have expected the Framingham subjects to have significantly lower insulin levels than Fukuoka subjects because their samples were collected earlier.

In conclusion, our overall data suggests that insulin levels are significantly lower in Japanese subjects than Caucasian subjects and that insulin deficiency may play a major role in causing excess diabetes in Japanese men. Our findings suggest that therapies that augment β cell function such as glucagon-like peptide-1 or dipeptidyl peptidase-4 inhibitors, but not sulfonylureas would be more effective in the management of diabetes among Japanese.

## Methods

### Study population and design

This study is a population-based, international cohort-comparative, cross-sectional design. The Japanese population consisted of a part of the Kyushu-Okinawa Population Study (KOPS), a community-based prospective observational study of cardiovascular disease (CVD) and its risk factors. KOPS has been underway since 2004 in four areas in Japan’s Kyushu Province including Okinawa and consists of 18,762 community-dwelling native Japanese women and men^25^,^26^. The participants of this analysis were notified, by local newspaper and public announcements, of a free annual health examination in 2007 given by the department of General Internal Medicine of Kyushu University Hospital. All of residents who agreed to participate in the study signed an informed consent form prior to enrollment. Data collected on each Japanese subjects included fasting blood sample, a dietary and lifestyle interview, and anthropometric measurements. In all individuals, height and weight were measured with light clothes and without shoes. BMI (kg/m^2^) was calculated as a measure of weight relative to height. The waist circumference was measured midway between the lowest rib and the iliac crest, in a standing position. To ensure the validity of the data, all doctors who participated in the study were staff members of the department of General Internal Medicine of Kyushu University Hospital who had been trained with regard to the study protocol and the medical procedures. KOPS was carried out in accordance with the principles of the Declaration of Helsinki as revised in 2008 and approved by the Kyushu University Hospital Ethics Committee.

American subjects were selected from participants in cycle 6 (1995 through 1998) of the Framingham Offspring Study, a long-term community-based prospective observational study of risk factors for CVD which consists of the offspring and their spouses of the original Framingham Heart Study cohort^27^. All participants executed written informed consent and the population is almost entirely Caucasian. For this analysis, we included female and male participants without currently using insulin or taking medications for cholesterol lowering or hormone replacement treatment from both populations. We selected 1,108 participants of KOPS (686 women and 422 men) and 1, 096 participants of Framingham Offspring Study (681 women and 415 men) matched with the Japanese subjects for sex, five-year age range, and (for women) menopausal status. Because sex was considered a confounding factor, those populations were divided into women and men as regards the statistical analyses. All studies were carried out in accordance with the principles of the Declaration of Helsinki as revised in 2008 and with the approval of the human investigation review committees of Boston University School of Medicine and Tufts University School of Medicine, Boston, MA, USA.

### Laboratory Measurements

Fasting plasma samples obtained from both Framingham and Japanese subjects were immediately stored at −80 degrees C and never thawed prior to use in this study and analyzed using the same assays in the same central laboratory at Tufts University. Plasma concentrations of GA and total albumin were measured using assays from Asahi Kasei (Tokyo, Japan) as previously described^25^,^26^. The GA assay had within- and between-run coefficients of variation of 1.1% and 1.6%, respectively. Plasma levels of adiponectin were measured using latex turbidimetric immunoassay kits obtained from Otsuka Pharmaceutical and Mitsubishi Chemical Medience (Tokyo, Japan). For the adiponectin assay, the within- and between-run coefficients of variation were 0.6% and 1.3%, respectively^28^.

Insulin levels were measured with an automated immunoassay obtained from Kamiya Biomedical (Seattle, WA, USA), as described previously^28^. For the insulin assay within and between-run coefficients of variation were 3.1% and 3.4%, respectively^28^. Fasting glucose levels were measured, as previously described^28^. The HOMA-IR, reflecting whole-body insulin resistance, was calculated using the following formula: [fasting plasma glucose level (mmol/L) × fasting insulin level (μiu/mL)]/22.5^29^. In addition HOMA-β was calculated with the following formula: [360 × fasting insulin level (μiu/mL)]/[fasting glucose (mmol/L) × 18–63]^30^. Both populations were stratified into NGT, IFG, or diabetes groups based on having a history of diabetes or fasting plasma glucose level using standard ADA criteria (fasting glucose: NGT < 5.6 mmol/L, IFG 5.6–6.9 mmol/L, and diabetes >6.9 mmol/L)^31^.

### Statistical analysis

Data are expressed as median values with 25th and 75th percentile values (interquartiles). Categorical variables are reported as frequencies and percentages. Univariate analyses were performed using Mann-Whitney U test to compare continuous variables between groups or Chi-square or Fisher’s exact test for categorical variables. We performed age- and BMI-adjusted multivariable logistic regression analyses to identify associations between the presence of diabetes (the outcome of interest) and other potential risk factors including fasting plasma insulin, adiponectin and GA. To select variables for these analyses, waist circumference was excluded because of its strong correlation with BMI. Fasting plasma glucose was also excluded because the levels were one of criteria to stratify subjects in this study. HOMA-IR, and HOMA-βwere excluded because their formulas include fasting plasma glucose and insulin, which are potential confounders. Results are expressed as odds ratios (OR) and their 95% confidence interval (CI). All statistical analyses were performed using SAS version 9.3 (SAS Institute Inc., Cary, NC, USA). A *P* value < 0.05 was considered significant.

## Additional Information

**How to cite this article**: Ikezaki, H. *et al*. Ethnic Differences in Glucose Homeostasis Markers between the Kyushu-Okinawa Population Study and the Framingham Offspring Study. *Sci. Rep.* **6**, 36725; doi: 10.1038/srep36725 (2016).

**Publisher’s note:** Springer Nature remains neutral with regard to jurisdictional claims in published maps and institutional affiliations.

## Figures and Tables

**Table 1:**
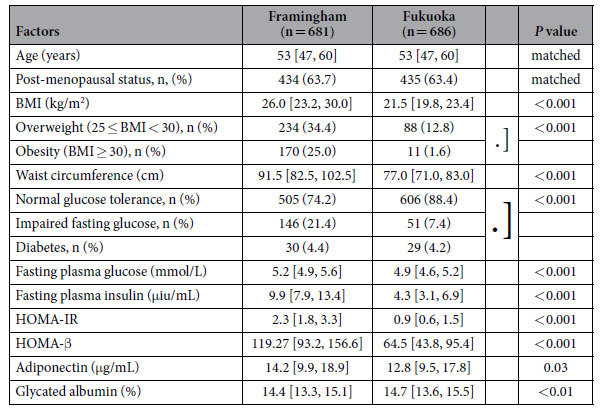
Parameters in Women*.

^*^Data are shown as median values [25% quartile, 75% quartile values] and number (%).

BMI, body mass index; HOMA-IR, homeostasis model assessment of insulin resistance; HOMA-β, homeostasis model assessment of β cell function.

**Table 2:**
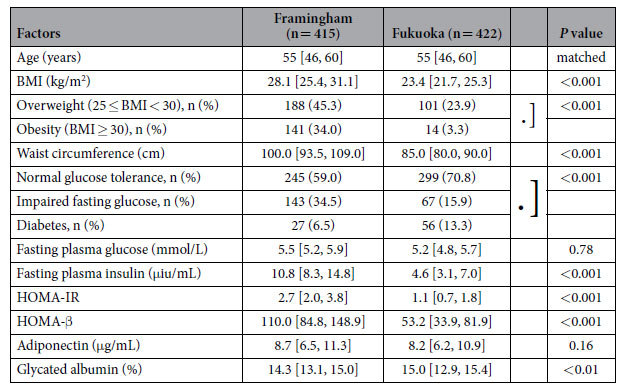
Parameters in Men*.

^*^Data are shown as median values [25% quartile, 75% quartile values] and number (%).

BMI, body mass index; HOMA-IR, homeostasis model assessment of insulin resistance; HOMA-β, homeostasis model assessment of β cell function.

**Table 3:**
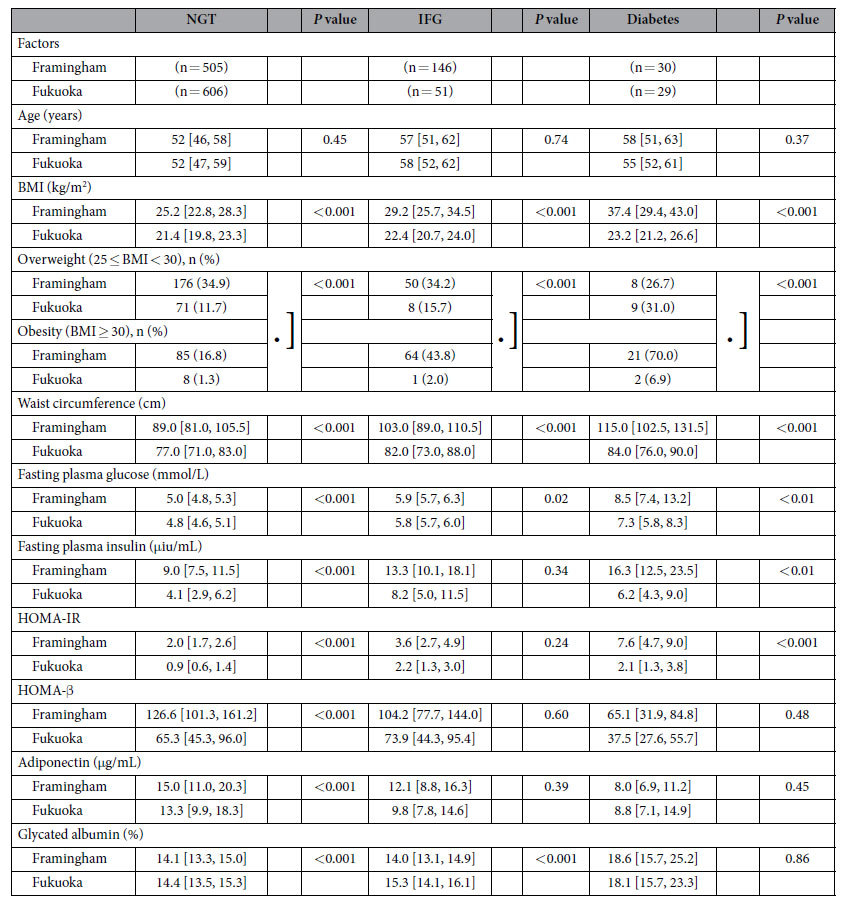
Parameters in women by glucose homeostasis*.

^*^Data are shown as median values [25% quartile, 75% quartile values] and number (%).

NGT, normal glucose tolerance; IFG, impaired fasting glucose; BMI, body mass index; HOMA-IR, homeostasis model assessment of insulin resistance; HOMA-β, homeostasis model assessment of β cell function.

**Table 4:**
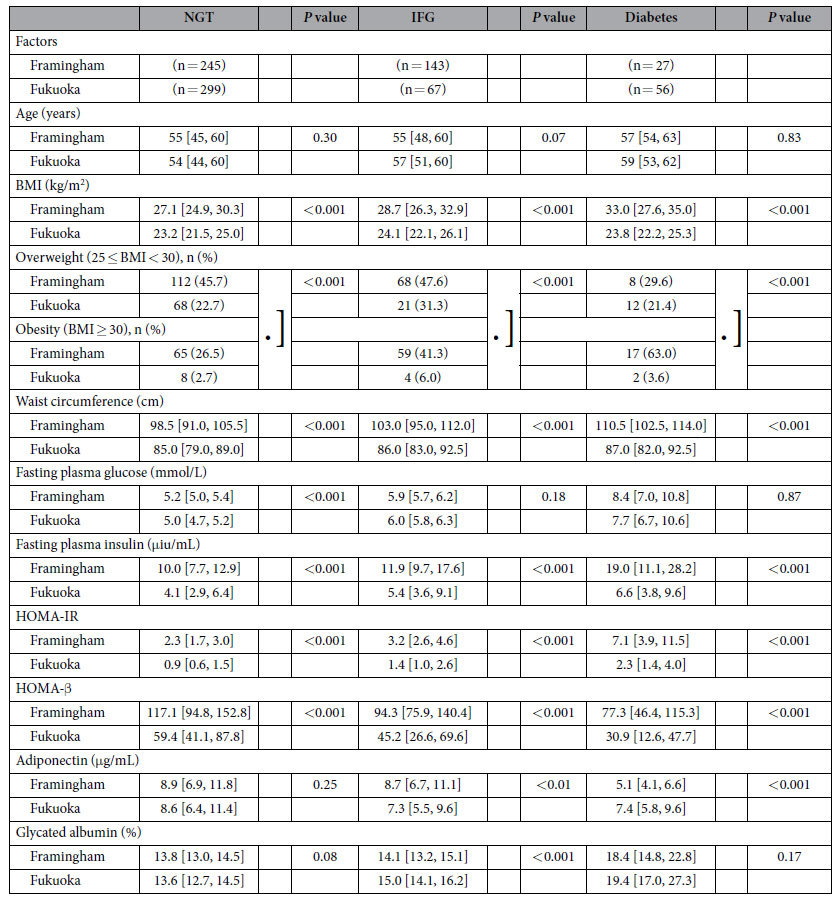
Parameters in men by glucose homeostasis*.

^*^Data are shown as median values [25% quartile, 75% quartile values] and number (%).

NGT, normal glucose tolerance; IFG, impaired fasting glucose; BMI, body mass index; HOMA-IR, homeostasis model assessment of insulin resistance; HOMA-β, homeostasis model assessment of β cell function.

**Table 5:** Age- and BMI-adjusted multivariable logistic regression analysis for presence of diabetes classified by sex and population (model 1).

Variables	Framingham		Fukuoka	
	OR (95% CI)	*P* value	OR (95% CI)	*P* value
Women				
Insulin (per 1 μiu/mL)	1.12 (1.00–1.25)	0.052	1.00 (0.96–1.03)	0.85
Adiponectin (per 1 μg/mL)	0.89 (0.77–1.04)	0.14	0.90 (0.81–1.00)	0.04
Glycated albumin (per 1%)	2.62 (1.88–3.65)	<0.001	2.18 (1.69–2.81)	<0.001
Men				
Insulin (per 1 μiu/mL)	1.03 (0.95–1.11)	0.49	1.11 (1.03–1.20)	<0.01
Adiponectin (per 1 μg/mL)	0.65 (0.49–0.86)	<0.01	0.97 (0.88–1.07)	0.51
Glycated albumin (per 1%)	1.74 (1.39–2.17)	<0.001	2.00 (1.65–2.42)	<0.001

BMI, body mass index; OR, odds ratio; CI, confidential interval.

**Table 6:** Age- and BMI-adjusted multivariable logistic regression analysis for presence of diabetes classified by sex and population (model 2).

Variables	Framingham		Fukuoka	
	OR (95% CI)	*P* value	OR (95% CI)	*P* value
Women				
Insulin (per 1 μiu/mL)	1.03 (0.96–1.10)	0.48	1.01 (0.98–1.03)	0.66
Adiponectin (per 1 μg/mL)	0.87 (0.77–0.97)	0.01	0.88 (0.81–0.97)	<0.01
Men				
Insulin (per 1 μiu/mL)	1.01 (0.95–1.07)	0.73	1.08 (1.02–1.15)	<0.01
Adiponectin (per 1 μg/mL)	0.61 (0.49–0.76)	<0.001	0.97 (0.90–1.05)	0.48

BMI, body mass index; OR, odds ratio; CI, confidential interval.
